# Gender and the performance of music

**DOI:** 10.3389/fpsyg.2014.00276

**Published:** 2014-04-16

**Authors:** Desmond C. Sergeant, Evangelos Himonides

**Affiliations:** Department of Culture, Communication and Media, International Music Education Research Centre, Institute of Education, University of LondonLondon, UK

**Keywords:** music, performance, musical meaning, gender, sex differences

## Abstract

This study evaluates propositions that have appeared in the literature that music phenomena are gendered. Were they present in the musical “message,” gendered qualities might be imparted at any of three stages of the music–communication interchange: the process of composition, its realization into sound by the performer, or imposed by the listener in the process of perception. The research was designed to obtain empirical evidence to enable evaluation of claims of the presence of gendering at these three stages. Three research hypotheses were identified and relevant literature of music behaviors and perception reviewed. New instruments of measurement were constructed to test the three hypotheses: (i) two listening sequences each containing 35 extracts from published recordings of compositions of the classical music repertoire, (ii) four “music characteristics” scales, with polarities defined by verbal descriptors designed to assess the dynamic and emotional valence of the musical extracts featured in the listening sequences. 69 musically-trained listeners listened to the two sequences and were asked to identify the sex of the performing artist of each musical extract; a second group of 23 listeners evaluated the extracts applying the four music characteristics scales. Results did not support claims that music structures are inherently gendered, nor proposals that performers impart their own-sex-specific qualities to the music. It is concluded that gendered properties are imposed subjectively by the listener, and these are primarily related to the tempo of the music.

## Three levels of music communication

Bertrand Russell said of language that “it expresses the state of the speaker and alters the state of the hearer” (1941, p. 204). He might well have made a similar observation about music, whose nature is “a form of communication in which all humans participate, analogous to language or speech” (Nettl, 2014) and whose purpose is “to please, and the pleasure consists in different emotions being activated in the listener” (Descartes, 1618).

Despite the comparability of their intentions, there are essential differences between speech and music in the nature of their significations. In speech, meanings attaching to words are relatively specific, at least to the extent of being capable of listing in a dictionary (Coker, 1972, p. 7) whereas those of music are less precise, though they cannot be regarded as being arbitrary (Monelle, 2000, p. 11). Seeger (1977) suggests that speech is communication of “the intellectualization of reality” whereas music is communication of “the feeling of reality.”

**Figure 1:**
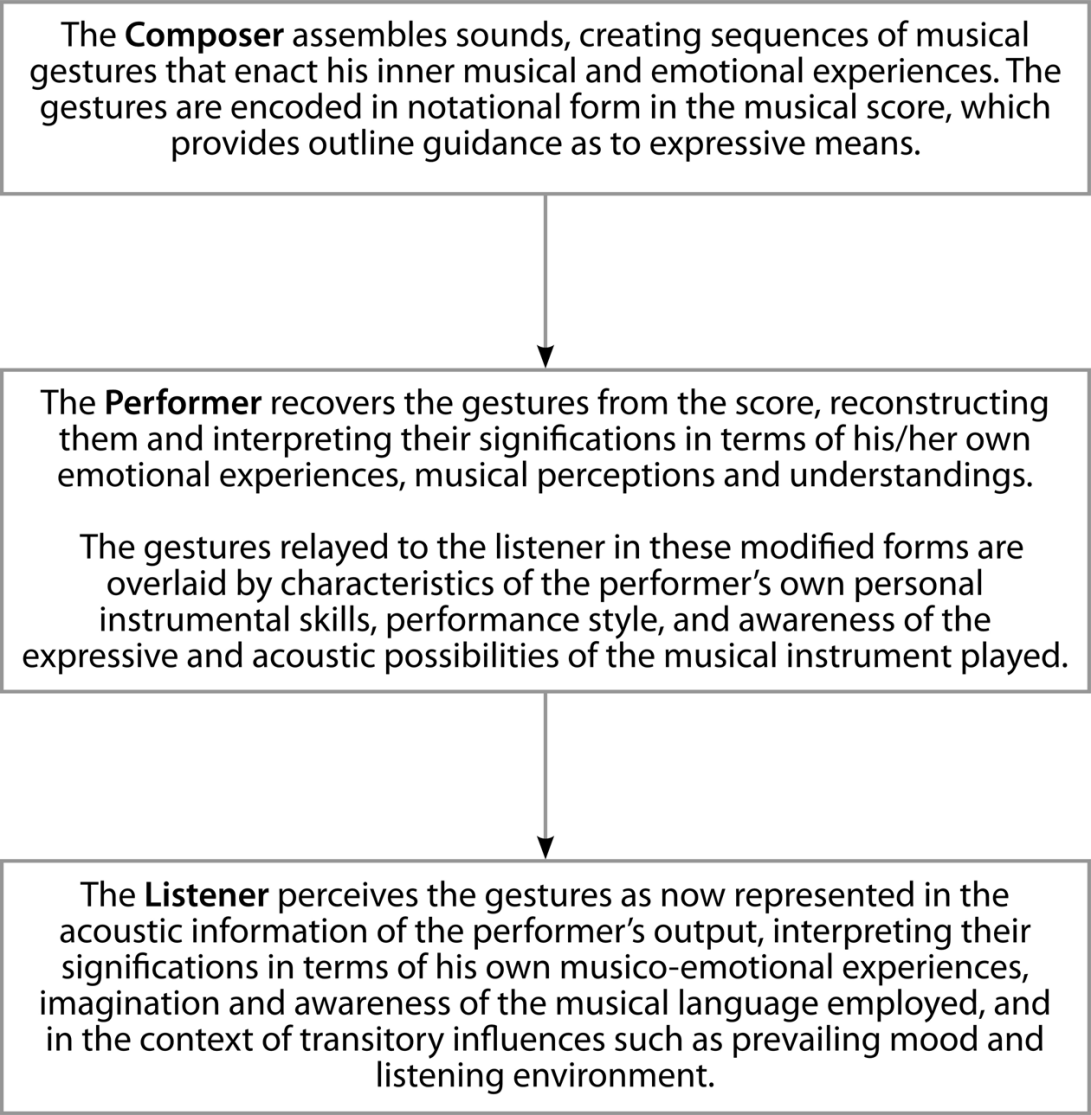
**The triadic transfer of music information**.

A second difference that distinguishes speech from music is that in the case of speech, the expression and consequent alteration of states take place through communication within a dyad—the speaker and the hearer—but in the Western “classical” art music praxis, the interchange of information passes through three levels. This triadic transfer can be modeled thus Figure 1.

The first generative level comprises the infrastructure of pitch, temporal and timbral events determined by the composer, intentional gestures organized in groups and sequences to form a musical narrative reflective of an inner mental and emotional state and, at least in the case of Western music, set out in the score as the givens of a composition (Kuhl, 2011; Katz, 2012, p. 171). The successful expression of meaning at this stage is reliant on the goodness of fit between the musical content of the gestural signs and the musical and emotional experiences of the composer at the moment of their conception and the accuracy of their notation in the score, i.e., their validity as expressions of thought (Thompson and Robitalle, 1992; Trevarthen et al., 2011).

Superimposed onto this structural data is a second level: that of the performer who actualizes the score into sound and “interprets” the compositional data, giving it expression^1^^,^^2^. The motivation in this is probably not so much a deliberate intention to make the music “expressive,” but more a desire to enable the listener to share the way the performer intrinsically feels “the music should go,” whilst being “truthful” to what are believed were the composer's intentions^3^.

The performer contributes to a “change of state” in the listener by means of small adjustments to parameters of tempo, phrasing, duration, articulation (staccato and legato), loudness, spectral envelope (timbre), dynamics and pitch (where the instrument of performance allows the player control of this), i.e., the *prosody* of the performance. Juslin (2001, 2003, 2009) provides detailed accounts and argues that no single dimension can encapsulate the expressivity of a musical performance: rather, the variables are highly interactive and operate variously in complex combinations. The degrees of freedom open to a performer to manipulate these parameters are not limitless: they are subject to stylistically accepted norms and median values, and to optimum values relative to each particular musical work (Holbrook and Arnand, 1990). Adjustments that are very small may be imperceptible to a listener: too great, and they are likely to be perceived as unacceptable exaggeration or waywardness, and departure from the “truth” of the composition.

The final level in the strata of communication is embodied in the listener, in reception of the sonic information and its neural transcription in processes of cognition and transformation into meaning. These essential processes are dependent upon the musical experience, awareness, and responsiveness of the listener and familiarity with the musical idiom of the composer. For the communication to be functional, the “meaning” imparted to a musical gesture by the performer must be recognized by the listener. Composer, performer, and listener must therefore understand a mutually shared code. Citron (1993) cautions that during a performance “each listener will be creating another ontology of the work. This newly constructed version probably does not coincide in all respects with the performer's understood version or with the composer's.” Sundberg (1993) similarly comments that “expressive details in music performance become meaningful because of the listener's extramusical experiences.” Although there is no certainty that the meaning intended by the composer is the same as that realized by the performer or understood by the listener at the final stage, there is good evidence that there will be at least positive correspondence (Krumhansl, 1997, 2000; Balkwill and Thompson, 1999; Juslin and Laukka, 2003, 2004; Juslin, 2005; Bhatara et al., 2011). Meaning in music is therefore not an absolute quality, and Pearsall and Almen (2006, p. 1) describe the term as having a degree of “semantic slipperiness.”

With three levels of information transfer in the musical communication process, it follows that three levels of musical meaning are operative. The first is, the “meaning” of the compositional events and structures, variously termed “embodied” or “intramusical” meaning (Meyer, 1961) and “inherent” meaning (Green, 1997), contained within the music itself, a view predicated on the concept of predictive meaning whereby a gesture may lead to expectancy of future gestures having similar or related characteristics (Coker, 1972, p. 5). The second level is the expressive meaning introduced at the performance level, and lastly that invoked in the listener. The apprehension and interpretation of meaning by the listener is subject to constraints of listener mind-set, social perceptions, availability of appropriate cognitive abilities, and experience of the musico-linguistic idiom employed (Dellacherie et al., 2011), a condition that accords with Lowery's definition of music as “… a subjective phenomenon that only begins when the sounds heard are understood” (1952, p. 18).

The problem of meaning in music has been the subject of extended discussion in the literature, and lies within the province of *semiotics*, the theory of signs and their significations. The process of semiosis is one by which a sign becomes a signifier for something else: its signification. “The communicator constructs an internal representation of some aspect of the world and then carries out some symbolic behavior intended to convey the content of that representation. The recipient must perceive the symbolic behavior and recover from it the content it signifies” (Juslin, 2005, p. 86). The adjustive response of the listener to a gesture therefore represents the meaning of that gesture, but the affective value of its signification is dependent on the contribution the perceiver brings to the event, and is therefore not inherent in it. Thus a knock at the door might constitute an objective sign, its signification being that a caller is outside, and the adaptive behavior that the door is opened. The affective value of the signification, however, would depend on the anticipation of the door's owner as to the probable identity of the caller: the postman, a lover, a debt-collector, the police!

## Gender and musical meaning

Given the human intentions and interactions intrinsic to this triad of stages of communication, it is not surprising that music has been argued to be a social phenomenon and that “musical meanings are socially and culturally constructed” (Olsson, 2007, p. 989), having “fundamentally a social life” (Feld, 1984), “growing out of specific social context, and expressing the assumptions of that context” (Citron, 1993, p. 120). Music is part of the constructs of our sense of identity within society (Frith, 1996, p. 124).

It has consequently been postulated that as social meanings are almost invariably gendered, so also are musical meanings. McClary (2002) for example, describes music as essentially “a gendered discourse” and the history of musical form as “a heavily gendered legacy” (p. 17), and considers that “classical music—no less than pop—is bound up with issues of gender” (p. 54). Treitler (2011) states that “… gender, race and ethnicity has the meaning,” and Taylor (2012) that “music is a dynamic mode of gender” and “a stylistic marker of sexual identity.” Hargreaves et al. (2005) list gender as a principal variable at each of the three stages of transfer of information: composer–performer–listener (pp. 8–15); but none of these writers provide an account of the modifications in musical infrastructure or output or perception by which gendering would be evidenced, nor whether these would be the same at each stage of information transfer.

Gender has been held to be inherent in basic music structures, and “reflected in perceptual and music phenomena” (Brett et al., 2006). Maus (1993) considers gender to be related to music theory, and by McClary as being present in resolution of chromaticism to the triad, thereby “taking on the cultural cast of femininity” (p. 124). Shepherd (1991) also interprets the inherent hierarchy of tonality as “an image of a gendered hierarchy of political and social hegemony,” and Green (1997) refers to “… the gender-related characteristics of the music itself” (p. 139).

Sexual dissonance is seen as embodied in the contrasting characteristics of first and second themes in sonata form (McClary, 2002, p. 124). This idea appears to have originated in A.B.Marx's music-theoretic text “*Die Lehre von musikalischen compositionen*” (1845), in the course of which he described second theme as being “of more tender nature, flexibly rather than emphatically constructed … as it were the *feminine* to the preceding *masculine*.” The analogy was repeated by Riemann (1888) and again by D'Indy (1909) and has subsequently become a common allusion, as has the application of the adjective *feminine* to a cadence that reaches closure on an unaccented beat^4^. Monelle (1995) describes the opening of the Tristan prelude as proceeding by “feminine quavers and dotted rhythm,” and Clément sees the chromaticism of the same passage as reflecting “seductive, deadly feminine sexuality.” Shepherd writes of “the role of timbre in articulating and reproducing gender identities,” “male timbres” and “female timbres” (1991, p. 170)

The association of music and gendering extends beyond gender to sexuality and sexual identity since “musicality is next-door to sexuality” (Cusick, 2006, p. 74) and “the history of Western music is a history of sexual anxiety, ambivalence and negotiation” (Peraino, 2003). For McClary, music is tied with the “channeling of desire … and competing images of sexuality,” and tonality to be “strongly informed by erotic imagery” (p. 54). Holsinger (2001) even sees female sexual desire and pleasure as inseparable from musical and devotional experience in the convent of Hildegard of Bingen.

The imputed gendering of musical signs becomes more complex when the connection moves beyond the boundaries of male/female heterosexuality: Cusick (2006, p. 67 et seq.) discusses the effects of “a Lesbian relationship with music” and Rycenga (2006) discusses “lesbian compositional processes” (p. 277) stating that being a lesbian “transforms the thought/action process that is composition” and Cusick speaks of a “Lesbian reception of music's message” (2006, p. 70). Brown (1986, pp. 100–187) sees strong associations between Tchaikowsky's fourth symphony op.36 and the composer's reported homosexuality.

Gender is repeatedly argued to be present at every stage of the communication triad: Green (1997, p. 71) cites the composer Ethel Smyth that “… she expected women to distinguish themselves from men not only in their approach and behavior, but also in their playing …” and argues that the listener receives a gendered message as “a cultural artifact within societal and historical context” (p. 6). A listener's sex, she argues, will “influence their overall response to music, and perception of masculinity or femininity, so that “… men and women must have a slightly different type of musical experience resulting from their gender” (p. 139). Citron (p. 120) also lists gender and sexuality among crucial factors for receptor.

Juslin (2005, p. 93) however, suggests that though present, their effects may be small “listener judgments are only marginally affected by musical training, age and gender of the listener,” and this view is shared by others (Gabrielsson and Juslin, 1996, 2003; Yamasaki, 2002). Biddleconbe (1992) has criticized McClary on grounds that she fails to explain how the codes of gendering might operate, and as Taruskin (1997) points out “if one is going to talk about a sign, one must also specify its referents.” Without hard evidence of the modus operandi by which composers and performers might imbue their musical output with gender implication, and that this is demonstrably received by listeners, linking signs with outcomes, these claims remain at the level of metaphor. Reference to *masculine* and *feminine* as descriptors of thematic characteristics or to *feminine endings* for cadence types may be helpful for illustrative purposes, but it requires a syllogistic leap to posit that they are therefore gendered in real musical experience. The diminished seventh chord has sometimes been described as the “Clapham Junction of tonality” because of its capability of resolution in multiple tonalities, but it has not been argued that at a point of its occurrence in a modulation a listener might therefore experience the feeling of the humdrum of a busy railway junction, nor in the field of language has the word *manhood* been argued to have feminine connotations because it is an example of a feminine ending.

Propositions of gendering therefore raise questions about the nature and processes of music communication, and of semiosis in music, and these can be rationalized into the form of three propositional hypotheses:

1. Compositional structures and the gestures they represent are inherently gendered, irrespective of era and style of composition;
2. Musical performances are conditioned by the sex of the performer in ways that are evident to appropriately skilled listeners;
3. Perception of musical meaning is qualitatively affected by the sex of the listener.

The extent to which these three propositions are supported by evidence of research is examined below. Areas of study in which relevant sex differences have been observed range across brain topography and neural functioning, language and communication, personality, emotionality, and expressiveness, musicality, and musical performance.

## Sex differences in communication and processing

### Sex differences in neural processing of music and music-related stimuli

Men and women do not differ only in physical attributes and functions: recent evidence from neurological research indicates that perceptual and cognitive processes are also sex-dimorphic, both in strength of activity and topographical deployment of brain resources. Kimura (2002) attributes this to differential effects of hormones which influence development of brain organization from very early stages of life, to the extent that the environment can be considered to be acting on differently wired brains.

Sex-specific contrasts in neural functioning have been shown in functional Magnetic Resonance Imaging (fMRI) studies to be evident in differential lateralization of brain behavior, with a greater level of bilateral functioning in females compared to more asymmetrical laterality in males (Koelsch et al., 2003a,b, 2005). Importantly, processing of auditory signals of both speech and music are affected by these differences (Brown, 1999; Gaab et al., 2003; Lattner et al., 2005; Ruytjens et al., 2007; Sergeant and Vraka, 2014). Recent studies have shown that auditory evoked potentials (AEPs) may differ in latency between sexes, with females showing significantly shorter inter-peak values measured at brainstem levels than males (Khatoon M Nighute and Ishaque, 2013). Auditory tympanometry indicators also show sex differences.

Inter-sex contrasts in electrophysiological measures indicating significant differences in brain reactivity have been observed by Nater et al. (2006): females showed greater sensitivity to aversive musical stimuli such as “heavy metal” and their data accord. Recent evidence from a study by Thorpe et al. (2012) suggest that neural sex differences may be reflected in differences in the ways that males and females process and predict musical structure (p. 459).

Notwithstanding this accumulating evidence of sex-related disparities, as Kimura (2002) remarks, unless differences in neural processing can be demonstrated to affect behavior in detectable sex-dimorphic ways, they are not meaningful in terms of human outcomes.

### Evidence of sex differences in comparable modes of communication: spoken language

The meaning of speech is not communicated solely by its semantic content: prosody (which includes the variables of pitch contour, average pitch level, intensity, tempo, duration, accentuation, average loudness level, and voice timbre) contributes importantly to communication of meaning by expressing emotional qualification of the semantic content, identifying important words and qualifying the category of an utterance as declarative or interrogative (Behrens, 1985; Besson et al., 2002; Mitchell et al., 2003; Sergeant and Welch, 2009). These complexes have been shown to be cross-cultural (Bolinger, 1978; Thompson and Balkwill, 2006) and represent a fundamental principle of human brain organization affecting both speech and music (Thompson et al., 2004, 2006; Nygaard and Queen, 2008).

Prosody can be considered to function independently of the semantic content (Frick, 1985; Standke, 1992; Banse and Scherer, 1996; Kitayama and Ishii, 2002; Ilie and Thompson, 2006). Schirmer et al. (2002) found behavioral and electrophysiological responses to prosody to be earlier in females than in males, for whom longer intervals between prime and target were required for processing. Similar findings are reported by Obleser et al. (2004); Koelsch et al. (2005) and Wurm et al. (2001), and are confirmed by an fMRI study by Imaizumi et al. (2004). Cortical areas for both words and prosody have been found to be activated significantly more strongly in females than in males, bringing the conclusion that there are sex-differentials in comprehension of emotional signals.

It is self-evident that men and women differ in the pitch range of speech, but the two sexes also differ in habitual speech styles and intonation patterns. Women's speech moves over a wider pitch range, and has greater dynamic flexibility, with more rapid pitch excursion, whereas men's speech is characterized by less dynamism (Daly and Warren, 2001). These differences have been interpreted as showing greater felt emotion and empathy in women (Fernald, 1989).

These studies indicate the presence of differential levels and styles of activity and responsiveness between males and females when engaging in communicative behaviors.

### Sex differences in personality characteristics, emotionality and expressiveness

It is a common-place postulation of gender stereotyping that the sexes differ in personality characteristics (Deaux and Lewis, 1984; Feingold, 1994; Banaji and Greenwald, 1995; Heiman, 2001). Women are commonly believed to be more emotional, more moody, more trusting, sympathetic, tender-hearted, community-spirited, more conservative in their use of language, and to show greater tendency to neuroticism (Hoffman, 1997). Men are viewed as more rational, assertive, agentic, less trusting.

In a qualified way, research evidence offers some support for these anecdotal wisdoms, but by no means unreservedly. The common stereotyping of women as more empathetic than men, is supported in the work of Trimmer et al. (1998), though this has been attributed to a greater social acceptability for women to display their emotions more overtly (Eisenberg and Lennon, 1983; Kelly, 1999). Feingold (1994) reports from a meta-analysis of research data that women are characterized by higher levels of extraversion and anxiety, are more trusting and especially are more tender-minded and nurturing, and this is supported by evidence of Baron-Cohen et al. (2005) of structural differences in the brain. Women are also observed to be more expressive of their emotions. Men are reported as higher on scales of assertiveness and self-esteem and lower on those characteristics observed in women. These sex differences are found to be constant across ages, years of data collection, and education.

Music preferences and responsiveness have been found to be reliably predicted by personality characteristics (Wheeler, 1985; Dollinger, 1993; Rentfrow and Gosling, 2003; Delsing et al., 2008; Zweigenghaft, 2008; Tekman, 2009; Luck et al., 2010; Langmeyer et al., 2012). Neuroticism has been shown to have a robust association with preference for classical music (Dunn et al., 2012) and “openness to experience” to be related to both a liking for jazz and a wider range of musical styles (Rawlings and Ciancarelli, 1997; Dunn et al., 2012) and to more intense experiencing of music-inspired emotions (Liljeström et al., 2012). A relevant constraint here, however, is that these findings have been drawn from broad general populations, and are unlikely to be consistent with evidence from musically trained subjects; Kemp's (1996) extensive study indicates that musicians are untypical in terms of measured personality, and are not conformant to patterns reported from general populations; a view that is supported by the work of Cutietta and McAllister (1997).

Sex differences have been noted in neural functioning in responses to music (Delsing et al., 2008; Lu et al., 2011), evidenced by increased activation in brain areas involved in auditory processing. This is consonant with findings of an fMRI study by Canli et al. (2002) that significantly more brain areas with faster response times were operative in women, leading to the conclusion that emotions were evoked more powerfully in females than males (Lin et al., 2010). Females are also reported to experience “chills” in response to music more than males (Panksepp, 1995).

### Gender and the performance of music

Although the studies reviewed above give some support to propositions that the sex of a performing artist might have some qualitative effect on the sonic qualities of that artist's performance. Evidence from analysis of actual musical performances is currently confined to a single study: Lehmann (2011) has reported analysis of 54 performances of the opening bars of “*Arlequin*” from Schumann's “*Carnaval*” op.9 extracted from commercially available recordings by renowned pianists, ten of whom were women. Weighting cases by sex of artist, female performers were found to perform the passage marginally, but statistically significantly, slower than the males (265 beats per min vs. 280 for males). However, as this study samples only the first eight bars of a single work its scale is too small to allow generalization to all musical literature or performance.

## Research design

The study reported below was designed to acquire empirical evidence to test the validity of claims of gendered properties of music and music performance, and to test empirically the three hypotheses. Musically experienced listeners, recruited via an internet facility, heard a sequence of extracts from recorded performances of music from the “art/classical” repertoire. At each extract they were asked to judge the sex of the artist performing—the “performer-sex discrimination task.” A second group of musically cognizant listeners listened to the same sequence of extracts, and rated the emotional valence of each item using four rating scales—the “music characteristics scales” and these provided data of the perceived emotional valence of each musical extract.

**Figure 2:**
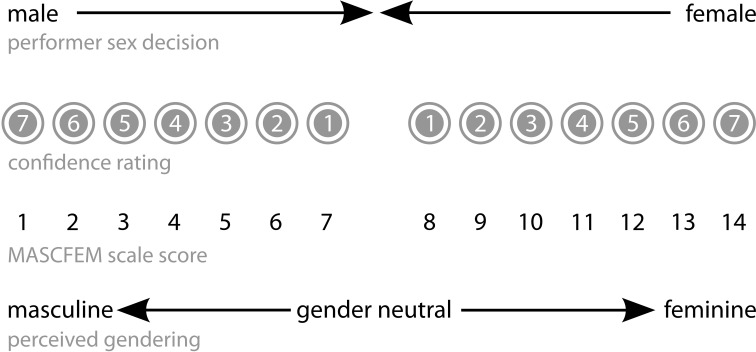
**Structure of the MASCFEM scale: perceived masculinity/ femininity of extracts and performances**.

A sensitive measure—the MASCFEM scale—was constructed from data generated in the performer-sex task by combining “*male/female*” performer-sex decisions with their associated confidence ratings. This provided a measure of perceived masculinity/femininity of the musical extracts, and facilitated examination of possible interactions between sex of performer and sex of listener. A decision that a performer was “*male*,” made with maximum confidence rating of 7 was taken as the optimal masculine polarity of the scale (low MASCFEM score = 1); similarly, a response of “*female*” made with a confidence rating of 7 was taken as the optimal feminine point (high MASCFEM score = 14). The mid-point of the scale (score 7–8) thus represented gender-neutrality (Figure 2).

Should sex-specific information be present at micro-levels of musical articulation of performances of male and female artists, they would be most likely to be detected by listeners who have themselves experienced sustained musical training and have acquired performance skills. Musically experienced listeners have, unsurprisingly, been shown to be superior to non-musicians in detection of small changes in pitch changes and pitch discrimination (Brattico et al., 2001; Tervaniemi et al., 2005; Fuller et al., 2014) pitch violations (Marques et al., 2007), beat perception (Grahn and Rowe, 2009), sensitivity to rubato (Johnson, 1996), tempo of orchestral excerpts (Geringer and Madsen, 1984, 2003), etc. These differences have been observed to reflect differences in patterns of brain behavior (Brattico et al., 2001; Onishi et al., 2001), and in times required for neural activity to reach peak responsiveness (Evers et al., 1999). In the event that musically skilled listeners were shown not to be able to differentiate performances of male and female artists at a statistically reliable level, it would be difficult to argue that sex-specific qualities were present in the musical information at a meaningful level.

## Materials and methods

### Performer-sex discrimination task

Two data-collection instruments (listening sequences) were constructed, each comprising 35 extracts taken from published recordings of music of the Western art/classical music repertoire. Extracts, each of approximately 1.5 min duration, were taken from either the opening bars of a composition or starting at an appropriate point later in its progress. In the case of ensemble works such as chamber or concerto movements (e.g., Mozart Clarinet Quintet), a passage where the solo instrument was prominent was selected.

The two sequences comprised extracts from the same musical works, starting and fading at identical points, the difference between them being that when the performer of an extract in one sequence was male, the performer of the matching extract in the other sequence was female, and vice-versa. With 35 extracts in each of the two listening sequences, male and female performances were sampled equally across 70 presentations.

In order to test the first hypothesis (gendered information is present in the structures irrespective of era and style of composition), works sampled (listed at Supplementary material) were selected from the standard repertoire so as to include a range of eras and genres of musical composition, ranging from smaller-scale pieces, and chamber works to solo concerti, such as might regularly be encountered by a music-listening audience. Instruments sampled in the performances were piano, harpsichord, violin, viola, cello, harp, trumpet, recorder, flute, oboe, clarinet, and bassoon. It would have been desirable that a greater range of brass and percussion instruments be included, but this was prevented by the limited availability of recordings of female performers of these instruments^5^ and therefore difficulty in finding matching female/male recordings of the same works. For obvious reasons, no vocal music was included. Works in major and minor modes and atonal examples were included.

Bearing in mind that era of composition may affect musical structure, meaning and interpretation (Christensen, 1995) and in order to test the second part of the first hypothesis, compositions sampled in the sequences ranged from early 18th century (earliest date of composition 1720, Handel suite no 5 HWV430) to mid-20th century (latest date 1911, Schoenberg, op.19). Hearing and responding to an entire Listening Sequence required approximately 56 min.

The great majority of composers have been male, and it would not have been possible to find sufficient recorded works by women composers to enable matching pairs of works in the sequences. None of the writers proposing gendering (cited above) however has suggested that gendering influences might be restricted to either sex, and almost all of the works to which gendering has been attributed by these writers have been works of male composers.

All digital audio files were scrutinized using a professional digital audio editing suite to ensure that no systematic differences of tempi (mean beats per min), pitch range, overall sound recording levels (means and peaks of loudness levels), or other acoustic factors were present between matched recordings of male and female performances. Any suspect files were replaced with other performances. Factors such as pitch range, timbre, would, of course, be common to both male and female performances of an extract.

#### Procedure

Listeners were advised in a preliminary “Guidance to listeners” paragraph that during the playing of an extract the name of the instrument to be judged would be highlighted on the response page, and that this was the instrument of which the sex of the performer was to be judged.

At each extract in the listening sequences, listeners were asked to judge whether the artist performing that extract was female or male. They were then asked to qualify their “*male/female*” response by rating their confidence in their decision using a 7-point scale where 1 = “*I'm not at all confident*” and 7 = “*I am very confident*.” After listening and responding, listeners were able to trigger the following extract when ready. It was possible to suspend the session for resumption at a later time, at which point the system restored them to the extract reached at the point of suspension.

The two sequences were made available to listeners via an online facility on the web server of the International Music Education Research Centre (iMerc) at the Institute of Education, University of London. When a potential listener logged on to the dedicated website, they were presented with an introductory page of information explaining the background, motives, and procedures of the research. When they had read to the end of the introduction, they were asked whether they wished to proceed to the Listening Sequence. On affirmative response they were randomly assigned by the automated system to either Sequence A or Sequence B. After completion of the sequence, the listener was asked to provide personal data of sex, age-group, instruments played and musical work-role. All responses were stored in the system for analysis.

#### Participants

A total of 138 listeners responded to the performer–sex identification task. 69 response sets were discarded, either because the listener had failed to listen to all 35 extracts and their response data were therefore incomplete, or in three cases, they had failed to provide details enabling them to be classified by musical experience, sex or age-group. The valid data sets therefore comprised 69 cases, 34 for Sequence A listeners, 35 for Sequence B.

### Music characteristics scaling

#### Materials and procedure

The two listening sequences used in the performer–sex discrimination task were evaluated further by a second group of listeners who had not participated as listeners in the performer-sex measures.

**Figure 3:**
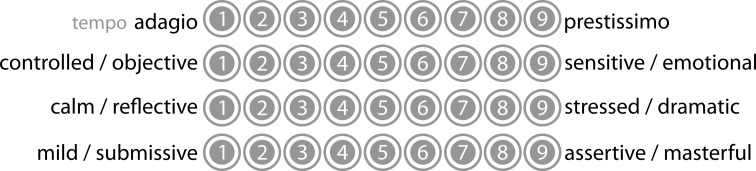
**Four “Music Characteristics” scales**.

These four additional measures were created in order to provide measures of perceived emotional valence of the musical extracts used in the listening sequences. Listeners for these measures were asked to rate each extract using 9-point semantic differential scales whose verbal polarities were designed to characterize the temporal density, emotional valence and mood of each extract (Figure 3).

#### Participants

All participants (*n* = 23) were graduate students following a master's degree course in music at a state university in U.S.A. All participants had extended advanced musical training. The task was completed using a bespoke designed online listening form/instrument.

## Results

### Performer-sex identifications

With 35 extracts in each sequence, and a two-alternative performer–sex response of “male” or “female,” the inherent probability of the measures was *p* = 0.5, giving an expected score at the binomial mean of 17.5. The range of scores that could be regarded as non-significant variants of the binomial mean would notionally extend between ±1.96σ (i.e., the point where *p* = 0.05 probability will commence), here ranging from a minimum score of 11.7 to a maximum at 23.3. Scores significantly removed from the mean would therefore lie below 12 or above 24.

The mean score for the combined A and B sequences obtained by the 69 listeners was 17.57, (17.53 for listeners to sequence A, 17.97 for listeners to sequence B, difference = 0.44, *t* = 0.60, *df* 67, *p* = 0.55, not significant), i.e., both located almost exactly at the binomial mean. It must therefore be concluded that, as a group, our listeners were not able to determine the sex of a performer, and this outcome applied to respondents to each of the “A” and “B” sequences.

The distribution of scores was slightly positively skewed about the mean. Two listeners recorded performer-sex decisions slightly above the upper expected upper limit of chance scores, both making 26/35 correct decisions. Given a total of 69 listeners, this would be within a number of borderline significant scores possibly gained by chance, and their performance is therefore not interpreted as indicating presence of reliable ability to identify the sex of a performer.

### Effects of sex of listener

Sex of listeners did not affect performer–sex decisions: the mean of correct decisions by male listeners was 17.45 and for female listeners 18.16, *t* = 0.91, *df* 63, *p* = 0.55, not significant. Responses to performances by male and female artists were examined by means of multiple regression analyses using the “backward entry” method, in order to identify possible effects of age-group, work-role and sex of listeners on judgments made for both male and female artists. None of these variables was found to have significant relationships with the dependent variables, and no tendency was found for female listeners to make superior judgments in respect of female performers, nor for male listeners to greater accuracy in respect of male performers (Table 1).

**Table 1:** **Effects of sex, work-role and age-group of listeners on performer-sex identifications**.

	**Male performers (F = 1.462, *df* 3, p = 0.234, n.s.)**	**Female performers (F = 0.363, *df* 3, p = 0.780, n.s.)**
Sex of listener	*t* = 0.813, *p* = 0.420, n.s.	*t* = 0.846, *p* = 0.401, n.s.
Age-group of listener	*t* = 1.385, *p* = 0.171, n.s.	*t* = 0.727, *p* = 0.470, n.s.
Work-role of listener	*t* = 1.447, *p* = 0.153, n.s.	*t* = 0.165, *p* = 0.869, n.s.

### Music characteristic ratings

Ratings for the 35 musical extracts on the four music characteristics scales showed remarkable consistency among the 23 listeners, with a mean inter-listener correlation of *r* = 0.545, *p* < 0.001. Of the 506 inter-listener correlations, only 12 failed to reach significance at *p* = 0.05 level, and most were significant *p* < 0.001. Between-sex differences in the application of the four scales by male and female listeners yielded non-significant *t* values. These results were taken as validation of the four scales as effective measures of musical affect.

The mean range of ratings was narrow for all four of the 9-point scales, extending over only 1.85 (*SD* = 0.427) for the “Tempo” scale, 2.04 (*SD* = 0.695) for the “controlled–objective / sensitive–emotional” scale, 2.37 (0.660) for the “calm–reflective / stressed–dramatic” scale and 2.23 (0.635) for “mild–submissive / assertive–masterful” scale. This reflected the high degree of agreement between these musician listeners. This was again taken as a validation of the scales as being meaningful verbal descriptors of real musical characteristics.

Ratings for three of the music characteristics scales were highly correlated (Table 2).

**Table 2:** **Correlations evident among ratings for the four music characteristics' scales**.

	**Controlled-objective / sensitive-emotional**	**Calm-reflective / stressed-dramatic**	**Mild-submissive / assertive-masterful**
Tempo: adagio-prestissimo	*r* = −0.659, *p* < 0.001	*r* = 0.702, *p* < 0.001	*r* = 0.737, *p* < 0.001
Controlled-objective / sensitive – emotional		*r* = 0.304 not sig.	*r* = −0.336, *p* < 0.05
Calm-reflective / stressed-dramatic			*r* = 0.941, *p* < 0.001

**Figure 4:**
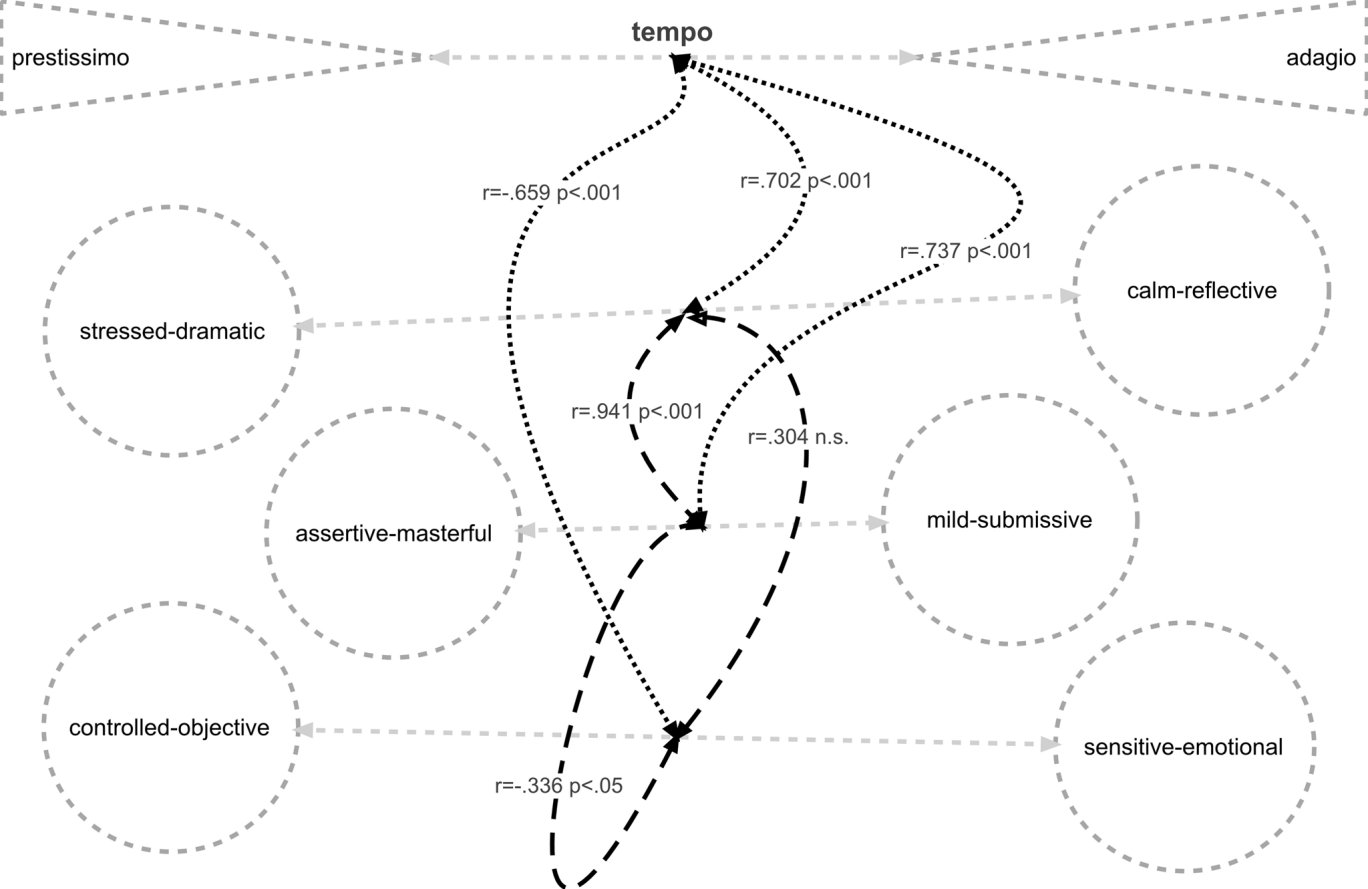
**Relationships of music characteristics to tempi**.

These intercorrelations reveal a somewhat complex relationship among the four dimensions (Figure 4), centered primarily on tempo: faster tempi were associated with perception of the music as more stressed and dramatic, more assertive and masterful, more controlled and objective: slower tempi were associated with music perceived as more calm and reflective, mild and submissive, sensitive and emotional.

When the inter-relationships of the four scales were subjected to multiple regression analyses independently of the dominant effects of fast or slow tempi, statistically significant associations were observed between

- “Tempo” and “mild–submissive / assertive–masterful” scales (*F* = 6.84, *df* 1.21, *p* < 0.02)
- “calm–reflective / stressed–dramatic” and “mild–submissive / assertive–masterful” (*F* = 27.14, *df* 2.21, *p* < 0.001),
- “controlled–objective / Sensitive–dramatic” and “mild–submissive / assertive–masterful” (*F* = 6.52, *df* 1.21, *p* < 0.02).

**Figure 5:**
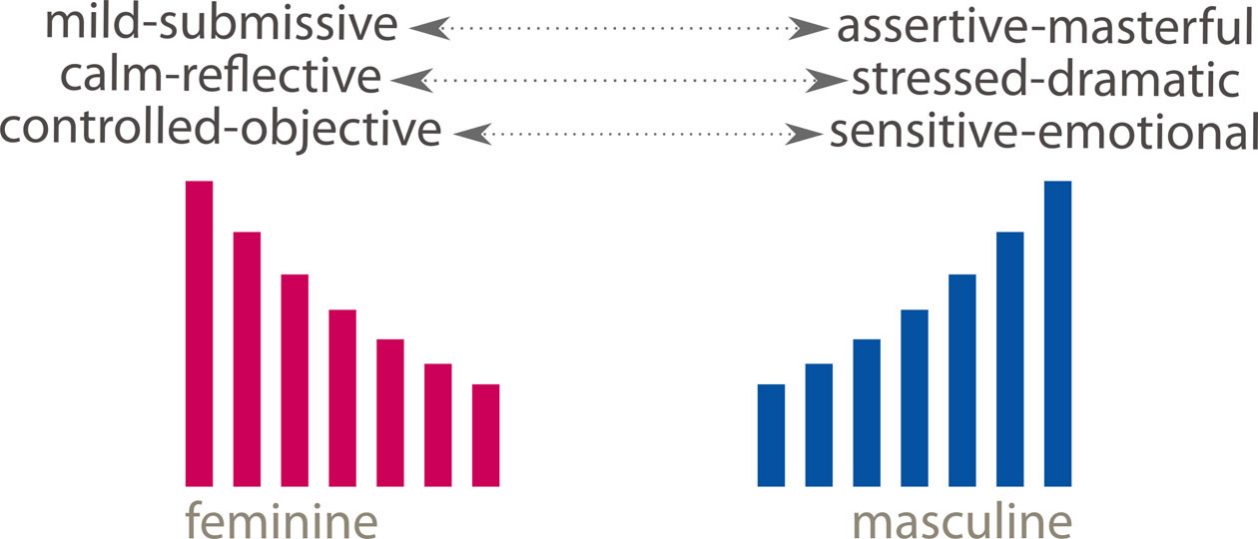
**Perceived Gendered propensities of music characteristics**.

MASCFEM scores for the combined A and B sequences were found to be significantly higher (i.e., more feminine) for extracts rated lower on the “controlled–objective / sensitive–emotional scale” (*t* = 2.69, *df* 68, *p* 0.009), the “calm–reflective/stressed–dramatic” scale (*t* = 1.98, *df* 68, *p* 0.05) and the “mild–submissive / assertive–masterful” scales (*t* = 2.00, *df* 68, *p* 0.05). The masculine / feminine directional polarities that emerge are shown at Figure 5.

### Effects of era of composition

Possible effects of era of composition on affect ratings of the musical extracts were examined by applying weightings to the extracts: extracts composed between 1650 and 1800 (era 1: baroque-classical) were assigned a weighting of 1, extracts composed between 1801 and 1900 (era 2: romantic) were weighted 2 and those composed between 1901 and 2000 (era 3: post-romantic-modern) were weighted 3. Ratings for the “controlled–objective / sensitive–emotional” scale were found to be related to the eras of composition of the musical extracts. Unsurprisingly, compositions of era 2, the so-called “era of Romanticism” were rated significantly higher on this scale than were compositions of the preceding and following compositional eras. Rating contrasts between compositional eras were: era 1 vs. era 2, *t* = 4.927, *df* 22, *p* < 0.001; Era 1 vs. era 3, *t* = 2.656, *df*. 20, *p* = 0.015, Era 2 (mean = 6.45) vs. era 3 (mean = 5.55) not significant.

Although these results indicate that differences in affect levels were recognized by the music-characteristic listeners, (music of era 1 judged to differ qualitatively from that of eras 2 and 3) neither accuracy of performer-sex decisions nor scores on the MASCFEM scales reached significance for any era. Our results therefore provide no evidence of qualitative differences between male and female performers; female artists are not identifiable by reason of any tendencies to emotionality stereotypically attributed to women, or empathy with affective characteristics of the music performed.

## Summary of findings

(1) Performer-sex discrimination: mean scores for the two performer-sex discrimination sequences were very close to their binomial means, indicating that as a group these musically experienced listeners were not able to determine the sex of a performer when restricted to auditory information.

Neither Sex, age-group nor musical work-role of listeners were found to be related to accuracy of performer-sex decisions. No superiority of decisions in respect of performances by own-sex artists was found.

(2) Music characteristic ratings by 23 musically trained listeners on the four scales showed a high level of consistency, with most inter-listener correlations reaching significance p<.001. Analysis of between-scale correlations revealed complex but systematic relationships among variables, with tempo as the controlling parameter.
(3) The MASCFEM scale enabled a more refined analysis of potentially gendered polarities in the judgments of listeners in the performer-sex task and possible relationships to music characteristics ratings. Gendered tendencies were noted but these were strongly related to tempo of the musical extract, and no relationship to the sex of performer was evident.
(4) Eras of composition: although ratings on the music-characteristic scales showed that listeners consistently identified differences in affect characteristics of music of the three eras of musical composition, this was not found to be related to performer-sex identifications, female artists were not found to reveal their sex by a detectably greater empathy for music rated as having greater emotional valence.

## Discussion and conclusions

Perception of gendered qualities by the listener at the final stage of the musical information transfer would necessarily lead to one of two conclusion: either the gendering originated at one of the preceding stages—composition, or performance—and was therefore present in the message as it reached the listener, or that it was a product of the listener's own cognitive constructs. In the latter case gendering would be a subjective property subscribed by the listener and could not be considered to be an inherent quality of the message.

In this context, the validity of the three propositions can each be evaluated from the results of the study.

Proposition 1: that musical structures inherently possesses characteristics that are gendered, irrespective of era and style of the music's composition.

Imposition of gendered information at the compositional stage would presumably require a deliberate intention on the part of the composer, but no indicative criteria have yet been proposed against which a composer could determine which sounds would be perceived as masculine and which would be feminine.

Of course, as McClary (2002, pp. 56–65) points out, male composers do sometimes write passages that are intentionally descriptive of feminine qualities, witness an example which she discusses at some length—the *habañera* sung by Carmen in the first act of the opera of that name by Georges Bizet, in which she taunts her would-be lover with promises of gratification “… this year, next year, sometime, never …”! Here the composer sets out to portray the sexual allure of the gipsy factory girl, as she flaunts her womanly attractions. Ballantine (1984, p. 52) similarly draws attention to the sexual ambiguity of the music at the point of the “double-blind” of cross-dressing and a soprano-voiced male character during the interaction between Susanna, Cherubino, and Countess Rosina in Act II of Mozart's opera *Le Nozze di Figaro* (see Keyser, 1987 for a commentary). Gendered musical intentions such as these, however, are particular to the genres of music-drama and dance music which according to Hanna “is embedded in divine sanction of sex and erotic fantasy” 1988, p. xvii), and there are no grounds for an assumption that because they exist in these specific situations they may therefore be considered to be equally present in the wider genre of instrumental music.

Proposition 1: results of the performer-sex discrimination task show that listeners do not recover gendered information from the musical signal as passed on by the performer. Unless it is argued to have been removed during the performance, it cannot be held to have been present in the compositional gestures set out by the composer. Proposition 1 therefore fails, and gendered information, if present in the signal, could only have been imparted at the stage of performance.

Proposition 2: that male and female performers imbue the overall musical message with qualities or characteristics unique to their own-performer-sex.

A minimum criterial requirement for validation of this proposition would be that appropriately musically experienced listeners should demonstrate a statistically reliable ability to determine which of the *male/female* polarities of the gendered information was represented in musical works they hear. Our 69 musically experienced listeners signally failed to demonstrate such ability: mean scores for identifications were very close to the binomial mean, and well within the range of chance. Male and female listeners were equally unsuccessful, and between-sex *t* tests proved non-significant.

We therefore conclude that any “change of state” experienced by our listeners was not conditioned by information relating to the sex of performer. The second proposition is therefore not substantive.

Proposition 3: that male and female listeners take away different features and “delineated gendered meanings” from the musical experience.

In so far as an ability to identify the sex of a performer can be considered an essential condition for presence of such gendered information, our listeners failed to provide evidence to support this third proposition. Performer-sex identifications made by male and female listeners in respect of both male and female artists all failed to rise above chance level. Regression analyses failed to yield significant *F* values for the sex, age-group or music work-role of listeners, and even the summed variances for these three variables made negligible contributions to the total variances. On prima facie evidence therefore the third proposition appears not to find support in our data.

Verbal descriptors can at best provide approximations to actual musical experience; nevertheless their use has long-enduring provenance as a means of measurement, (see Asmus, 2009, for an extended review). The 23 musically trained listeners rated the 35 extracts of the listening sequences against four “Music Characteristics” scales whose qualifiers were descriptors of qualities inherent in the music itself, not of the performances. The listeners were assigned by the online system randomly to sequences A and B, in the manner previously described, so that differences and correlations observed among the data therefore derive from performances by artists of both sexes. The four scales yielded very concise data that showed remarkable consistency. This persuades us that the semantic differentials employed to define the polarities of the scales were valid as qualities that musicians recognized as present in the musical extracts they heard.

Three of the scales were found to be highly correlated, creating a triadic relationship, with tempo as its pivotal quality. This is conformant with well-rehearsed evidence: early studies (Hevner, 1936, 1937, p. 625; Rigg, 1940) had observed that speed was a primary determinant of musical mood and listener response, and that variations in tempo invoked strong effects on affective responses. Budd (1985), similarly describes tempo as an “essential musical device for expression of emotion,” and a critical factor of musical interpretation. Other studies have yielded similar findings (e.g., Brown, 1979, p. 32; Duerr, 1981; Geringer and Madsen, 1984, 2003; Holbrook and Arnand, 1990; Gabrielsson and Lindström, 1995; Sheldon and Gregory, 1997; Gagnon and Peretz, 2003; Webster and Weir, 2005).

Ratings on the “controlled–objective vs. sensitive-emotional” scale were found to differ between musical works composed during the three compositional eras, 1650–1800, 1801–1900, and 1901–2000.

The MASCFEM scale enabled measurement of masculinity/femininity tendencies in the “Music Characteristics” ratings. Since the Performer–sex task listeners were a separate group from those generating the “Music Characteristics” ratings, data from the two sets of judgments were independent, and comparisons of the two scales are free from cross-contamination.

The results of this analysis run counter to stereotypical expectations: on the basis of much-repeated wisdoms, partly supported by research evidence, it might have been anticipated that greater emotionality and “sensitive-dramatic” polarity adjudged to be present in some music extracts would have been rated more highly toward the feminine polarity of the “MASCFEM” scale. The opposite was the case: MASCFEM scores were higher (i.e., more feminine) for extracts that were judged to be more “controlled-objective,” “calm-reflective” and “mild-submissive.” This effect may possibly be related to Kemp's (1996) observations that personality traits of musicians tend to run counter to trends observed for normal population samples.

Our conclusions from this study are that (i) except in special situations such as those discussed by McClary and Ballantine, music structures, at the point of composition, are innocent of gender implication, and that gendering is not an inherent quality of music structure, *per se*; (ii) our data do not support claims that performers impart their own sex-specific information into the sonic soundscape of their musical performances. The evidence of the inter-correlations between ratings of the four music characteristics scales (Figure 4) and the polarities of emotional valence indicated by application of the MASCFEM scale (Figure 5), however, suggest that gender-related perceptions may be understood by listeners. We conclude that if gendered perception of music is a reality, the perceived properties are subjectively imposed on the musical message by the listener,” through the listener's appropriations” (DeNora, 2000) and are primarily related to the tempo of the music.

### Conflict of interest statement

The authors declare that the research was conducted in the absence of any commercial or financial relationships that could be construed as a potential conflict of interest.

## Footnotes

^1^At an early stage in the development of music computing, when reliable sound synthesis first became a reality, a forthcoming radio programme was announced which was to include a computer-generated performance of a work by J. S. Bach. The news was greeted with keen anticipation by a sector of the press; it was seen by press music critics as heralding the dawn of a new age for music performance. Here was a new means of flawless performance: not only would it be wholly error-free, it would be liberated from those personal vagaries and idiosyncrasies of a human performer that were held to mask the composers true intentions. A computer-based performance could thus bring direct and definitive insights into the composer's mind. Retrospective commentary after the broadcast, whilst acknowledging the event to have been a significant engineering achievement, generally recognized that, as a musical experience, the performance had disappointed. It had seemed lifeless, dull and mechanical, and the anticipated insights had not been realized. The event served as a useful reminder that it is those very human vagaries and idiosyncrasies that release a musical work from the constraints of the otherwise ‘dead’ printed score (Goebl et al., 2007) and give it expressive meaning, that “the letter killeth, but the spirit giveth life” (St. Paul; Campbell, 2010). As Sloboda (1986) comments, if technical mastery and accuracy of tone and timing are all there are to music performance, we may just as well pass over the task to computer programmers and close our conservatoires.

^2^“Even if I am not a creative artist, still I am recreating” (Clara Schumann, as cited in Reich, 1985).

^3^The performer “must play everything in such a way that he himself will be moved by it.” Leopold Mozart, cited by (Strunk, 1950).

^4^The expression “feminine ending” is not unique to music, it is also used in the fields of linguistics and literature to describe a two-phoneme rhyme of which the penultimate is stressed but the final unstressed, (“*comment*,” “*gravel*” “*torrent*”), whereas in a masculine rhyme the stress is on the final syllable of the words (*restore*, *sublime*).

^5^The “Red Catalogue” listing all published recordings in CD format for the year in question listed no solo recordings of women players of French horn, trombone, tuba or percussion (Red Catalogue, 2004).
