# Supplementary material for: Gender and the performance of music
Source: Front Psychol. 2014 Apr 16;5:276. doi: 10.3389/fpsyg.2014.00276 (PMC3997045; doi:10.3389/fpsyg.2014.00276)
Supplement: Supplementary file 1 [file Presentation1.PDF]

Appendix 1

Music repertoire and performing artists used in the listening sequences.

**Extract**	**Sequence A**	**Sequence B**
Brahms: E min ‘cello sonata	Mistlav Rostropovich	Jaqueline Du Pré
Handel : Harmonious Blacksmith	Sophie Yates	Robert Aldwinkle
Eben: Chagal Red Window	Alison Balsom	Harry Kvebæk
Haydn: ‘cello concerto C major	Jean-Guihen Queyras	Maria Kliegel
J.S.Bach: 2pt invention A minor	Angela Hewitt	Evgeny Korolov
Rachmaninov: Paganini Rhapsody	Philippe Entrement	Valentina Kamenikova
Mozart: Oboe Quartet	Gordon Hunt	Sarah Watkins
J.S.Bach: ‘cello suite G major	Ophélie Gaillard	Pierre Fournier
Brahms: Clarinet sonata Eb major	Michael Collins	Thea King
Mendelssohn: Violin Concerto	Victoria Mullova	Stefan Jackiw
Rachmaninov: 3rd Piano Concerto	Martha Argerich	Zoltan Kocsis
Shostakovich: Violin Concerto	Maxim Vengerov	Lydia Mordkovitch
Vaughan Williams: Lark Ascending	Iona Brown	David Nolan
Dacquin: Le Coucou	Sphie Yates	Robert Aldwinkle
Fauré : Impromptu	Katherine Stott	Pascal Rogé
Mozart: Bassoon Concerto	Michael Chapman	Julie Andrews
Hasselmans: La Source	Marcus Klinko	Rachel Masters
Saint-Saens : Le Cygne	Maria Kliegel	Christopher v.Kampen
Beethoven : Sonata op.14	Alfred Brendel	Annie Fischer
Debussy: Syrinx	Gunilla v. Bahr	Michael Cox
Massenet: Mediatation Thaïs	Lorraine McAslan	Nigel Kennedy

Gershwin : ‘I got Rhythm’	Nigel Clayton	Angela Browning
Debussy: Reflets dans l’eau	Francois-Joel Thiollier	Noriko Ogawa
Stamitz : Viola Concerto D major	Tabea Zimmerman	Jan Pěruška
Saint-Saens: Romance	William Bennet	Susan Milan
Mozart: Clarinet Quintet	Andrew Marriner	Joy Farrall
Tchaikowsky: Pno Cto Bb minor	Emil Gilels	MarthaArgerich
Finzi: Bagatelle	Thea King	Robert Plane
J.S.Bach: Violin Concerto E major	Simon Standage	Catherine Mackintosh
Marcello: Oboe Concerto	Clare Shanks	Nicholas Daniel
Grieg: Piano Concerto A minor	Philippe Entrement	Eva Knardahl
Messaien: Le Merle Noir	Patrick Gallois	Susan Milan
Sammartini : Recorder Concerto	Konran Steinmann	Michaela Petri
Schoenberg: 5 little pieces op.19	Katharina Wolpe	Maurizio Pollini
Chopin: Scherzo C# minor	Christina Ortiz	Daniel Adni

trends observed for normal population samples.
